# Digital epidemiology and public health surveillance: scientometric mapping of emerging technologies and challenges (2000–2025)

**DOI:** 10.3389/fdgth.2026.1789164

**Published:** 2026-06-17

**Authors:** Génesis Carolina Arévalo-Guadalupe, Joaquin Estalin Gamboa-Tama, Katiuska Mederos-Mollineda, Dennis Alfredo Peralta-Gamboa

**Affiliations:** State University of Milagro, Milagro, Ecuador

**Keywords:** algorithmic ethics, digital inequity, global health, interoperability, scientific cooperation

## Abstract

The rapid advancement of digital technologies has transformed traditional mechanisms of epidemiological surveillance and response, giving rise to an interdisciplinary field known as digital epidemiology. This study presents a scientometric mapping of the main trends, collaboration networks, and thematic foci linked to the use of emerging technologies—such as artificial intelligence, machine learning, big data, the Internet of Things (IoT), and social media mining—in global public health surveillance from 2000 to 2025. The methodology combines bibliometric and social network analysis on documents indexed in Scopus and Web of Science, complemented by a qualitative examination of the fifty most cited studies. The results reveal an exponential growth in scientific output starting from 2010, with an inflection point during the COVID-19 pandemic. The United States, the United Kingdom, and China stand out as the primary centers of production and international collaboration. However, significant gaps persist in digital equity, interoperability, and ethical governance, particularly in regions with lower technological infrastructure. This work contributes to understanding the evolution and challenges of digital epidemiology, proposing a future research agenda centered on algorithmic ethics, transparency, international cooperation, and technological inclusion in vulnerable contexts.

## 1 Introduction

The 21st century has marked a profound transformation in the way societies record, process, and utilize health information. The convergence of artificial intelligence (AI), big data analytics, social media mining, the Internet of Things (IoT), and mobile platforms has driven the emergence of what is known as digital epidemiology, a field that integrates data sciences with public health to anticipate and manage health risks in real time (1, 2). This new paradigm represents a break from conventional epidemiological models, enabling the massive collection of heterogeneous data—clinical, environmental, and social—from multiple interconnected sources.

During the first two decades of the century, the expansion of global connectivity and access to digital infrastructures fostered the development of intelligent systems for epidemiological monitoring. However, it was the COVID-19 pandemic that solidified digitalization as a structural component of health responses. The global crisis highlighted both the potential of digital tools—capable of detecting early outbreaks, modeling disease spread, and coordinating interventions—and their associated vulnerabilities related to privacy, technological inequality, and data reliability (3, 4).

In this context, the present study aims to analyze the scientific and technological evolution of digital epidemiology through a mixed-methods approach that combines bibliometric analysis and qualitative review. The objective is to map research trends, international collaboration flows, the most influential publication sources, and the methodological, ethical, and structural challenges facing this field. Unlike previous studies focused exclusively on the impact of AI or big data, this research encompasses a broader spectrum of emerging technologies and integrates scientific performance indicators with perspectives on equity and digital governance.

In recent decades, the rapid expansion of digital technologies has profoundly transformed the landscape of public health surveillance. The integration of artificial intelligence (AI), big data analytics, social media mining, and the Internet of Things (IoT) has enabled the emergence of digital epidemiology as a novel paradigm for real-time monitoring and response to health threats (1, 2).

Despite its growing relevance, the literature on digital epidemiology remains fragmented across disciplines such as health informatics, data science, and epidemiology. Existing studies tend to focus on specific technologies—such as AI or social media analytics—without offering a comprehensive view of how these tools interact within broader surveillance systems. Furthermore, limited attention has been given to structural challenges, including interoperability, ethical governance, and global inequalities in digital infrastructure (5, 6).

Another critical gap lies in the lack of integrated analyses combining quantitative scientometric mapping with qualitative synthesis of influential studies. While bibliometric studies have documented publication trends and collaboration networks, they often lack interpretive depth. Conversely, narrative reviews provide conceptual insights but do not capture the structural evolution of the field at scale.

In this context, the present study aims to analyze the scientific evolution of digital epidemiology and public health surveillance between 2000 and 2025. Specifically, it seeks to (i) map global research trends and collaboration networks, (ii) identify dominant thematic areas, and (iii) critically examine technological, ethical, and structural challenges through the analysis of highly cited studies.

By integrating bibliometric techniques with qualitative analysis, this study contributes to bridging the gap between descriptive mapping and critical interpretation, offering a more comprehensive understanding of the opportunities and limitations shaping digital epidemiology as a global public health paradigm.

## 2 Methodology

The present study adopts a scientometric and qualitative design of a descriptive and exploratory nature, aimed at analyzing the global landscape of research on digital epidemiology and public health surveillance during the period 2000–2025.

A mixed-methods approach was employed, combining quantitative bibliometric and network analysis methods with a qualitative review of the most influential articles, with the purpose of identifying the main emerging technologies, predominant theoretical approaches, and the ethical and operational challenges associated with the digitalization of health surveillance.

### 2.1 Sources of information and search strategy

The collection of scientific data was conducted from the Scopus and Web of Science (WoS) databases, selected for their international coverage and the quality of their bibliographic records in health sciences, technology, and innovation.

The Web of Science collections included Science Citation Index Expanded (SCI-EXPANDED), Social Sciences Citation Index (SSCI), and Emerging Sources Citation Index (ESCI), to ensure multidisciplinarity and comparative validity of the records.

The search spanned the years 2000–2025, a period that coincides with the consolidation of digital health, the expansion of online epidemiological surveillance platforms, and the adoption of advanced analytics for outbreak and health emergency management.

The search equations were designed using Boolean operators and controlled terms that reflect the relationship between digital epidemiology, health surveillance, and emerging technologies, applied to the fields of title, abstract, and keywords:

Scopus: TITLE-ABS-KEY(“digital epidemiology” OR “digital surveillance” OR “infodemiology” OR “health informatics” OR “public health surveillance” OR “disease monitoring” OR “epidemic intelligence”) AND TITLE-ABS-KEY(“artificial intelligence” OR “machine learning” OR “big data” OR “social media” OR “biosensor” OR “mobile health” OR “genomic surveillance”)

Web of Science (WoS): TS = (“digital epidemiology” OR “digital surveillance” OR “infodemiology” OR “health informatics” OR “public health surveillance” OR “epidemic intelligence”) AND TS = (“artificial intelligence” OR “machine learning” OR “big data” OR “social media” OR “biosensor” OR “mobile health” OR “genomic surveillance”)

Scopus and Web of Science were selected as the sole data sources because they provide broad international coverage, high-quality bibliographic metadata, and strong multidisciplinary indexing in public health, medical informatics, digital health, and technological innovation. Their combined use is common in scientometric studies because it improves record reliability, facilitates citation-based analyses, and allows cross-database comparison while reducing the risk of relying on a single indexing system. Although other sources such as PubMed, Dimensions, or regional repositories may contain additional records, the decision to focus on Scopus and Web of Science was based on their suitability for bibliometric standardization, citation tracing, and international comparability.

### 2.2 Inclusion and exclusion criteria

Inclusion Criteria: Original articles, peer-reviewed, published between 2000 and 2025. Documents written in English or Spanish with full-text access.

Exclusion Criteria: Non-peer-reviewed works (e.g., preprints or conference proceedings). Duplicate publications between databases.

After applying these criteria, only records that explicitly addressed the application of digital tools in health surveillance, outbreak prediction, signal detection, or real-time epidemiological information analysis were retained.

### 2.3 Screening and selection process

The screening process followed a structured multi-stage procedure to ensure transparency and replicability. First, all retrieved records from Scopus and Web of Science were merged into a unified dataset. Duplicate detection was conducted using title normalization (lowercase transformation) and exact matching procedures in R, resulting in the removal of 780 duplicate records.

Second, a preliminary screening based on titles and abstracts was performed to exclude studies not directly related to digital epidemiology or public health surveillance. This stage led to the exclusion of 491 documents that did not meet the thematic criteria.

Third, full-text eligibility assessment was conducted on the remaining records. Studies focusing exclusively on bibliometric methods without substantive application to digital epidemiology were excluded (*n* = 10), ensuring that the final corpus reflected applied and conceptual contributions to the field.

**Figure 1:**
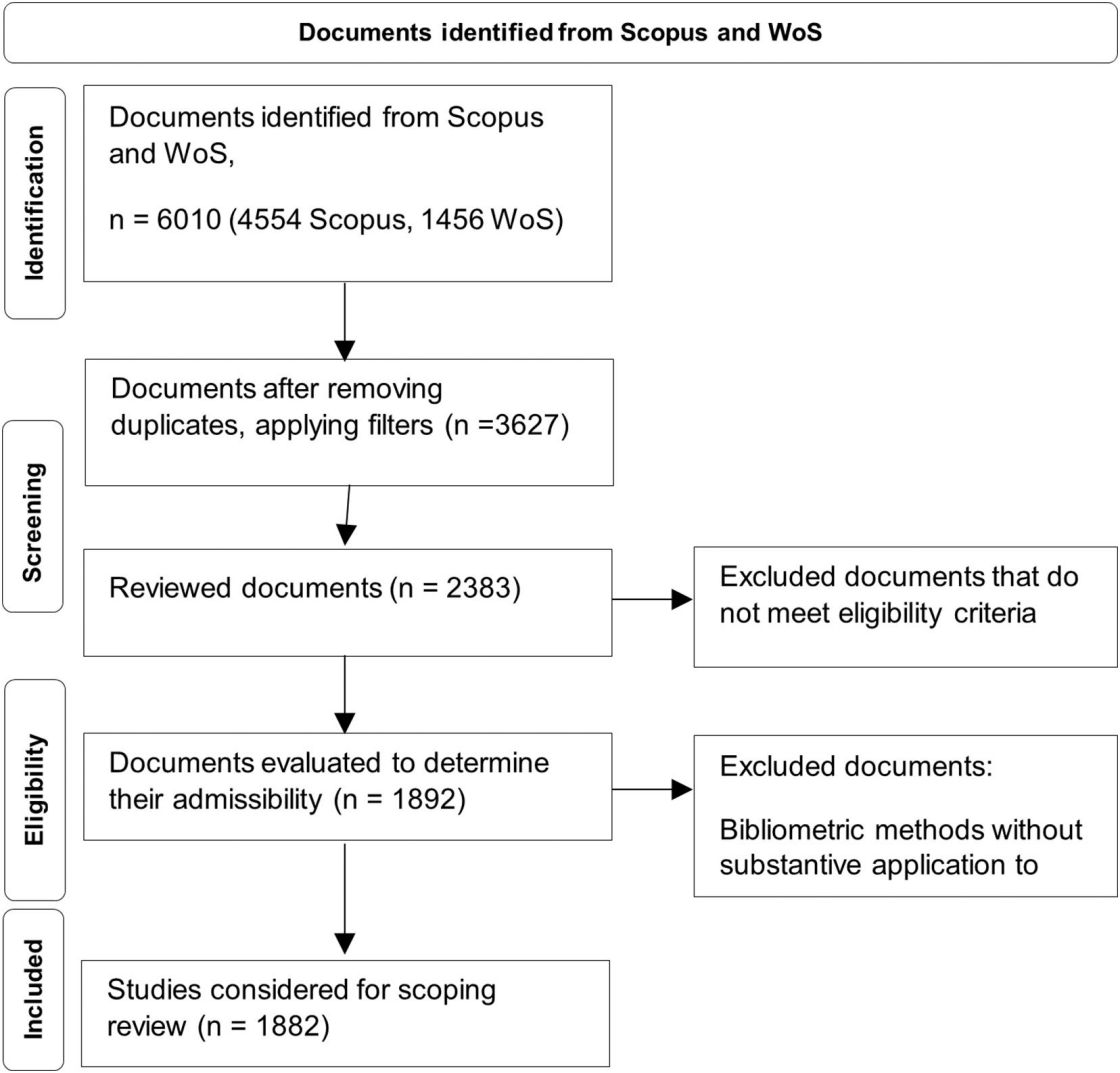
Flowchart of the PRISMA selection process.

Finally, 1882 documents were selected after reading the full articles (View Figure 1).

The screening process was conducted by two reviewers. In the first stage, both reviewers independently assessed titles and abstracts according to the predefined inclusion and exclusion criteria. In the second stage, full-text eligibility was evaluated independently. Any discrepancies were resolved through discussion and consensus, which helped reduce selection bias and strengthen methodological rigor.

### 2.4 Data cleaning and processing

Data cleaning procedures were implemented in R (version 4.4.2) using the libraries dplyr, data.table, and readxl. Text normalization was applied to harmonize author names and titles. Additionally, keyword filtering was conducted using the grepl() function to retain records containing relevant terms such as ‘digital epidemiology’, ‘health informatics’, and ‘epidemic intelligence’.

### 2.5 Analytical procedure

The analysis was conducted in three sequential stages:

- Descriptive bibliometric analysis: evaluation of annual production, citation patterns, and leading publication sources.
- Network analysis: construction of co-authorship and international collaboration networks using VOSviewer (version 1.6.20).
- Thematic analysis: keyword co-occurrence mapping to identify dominant research clusters.

Complementarily, a qualitative analysis of the 50 most cited articles was performed to examine technological scope, effectiveness, ethical considerations, equity, and implementation barriers. This mixed-method approach allows for both structural mapping and interpretive depth.

### 2.6 Bibliometric analysis

The analysis was conducted using R (version 4.4.2) and VOSviewer (version 1.6.20). The following libraries were employed: ggplot2 and gridExtra for representing the annual evolution of publications and citations. dplyr, tidyverse for identifying the most influential journals. openxlsx for exporting consolidated databases.

Keyword co-occurrence mapping, co-authorship networks, and collaboration were performed with VOSviewer, allowing the identification of thematic cores related to genomic surveillance, AI applied to outbreaks, use of social media data (Twitter, Google Trends), and integration of biosensors and mHealth platforms.

### 2.7 Qualitative analysis

For the qualitative phase, the 50 most cited studies within the final corpus were selected as a purposive subsample of high-impact publications. Citation count was used as the primary criterion because it allows identification of studies with strong scientific visibility and influence in shaping the field. However, the selection was not intended to represent the full diversity of the literature; rather, it aimed to capture the most influential contributions in terms of conceptual development, technological applications, and ethical debate. This strategy provides interpretive depth, although it may underrepresent newer studies with lower citation accumulation.

In addition to citation volume, thematic relevance to digital epidemiology and public health surveillance was verified during the qualitative review to ensure consistency between citation impact and substantive contribution to the study objectives.

## 3 Results

### 3.1 Analysis of productivity and citations

**Figure 2:**
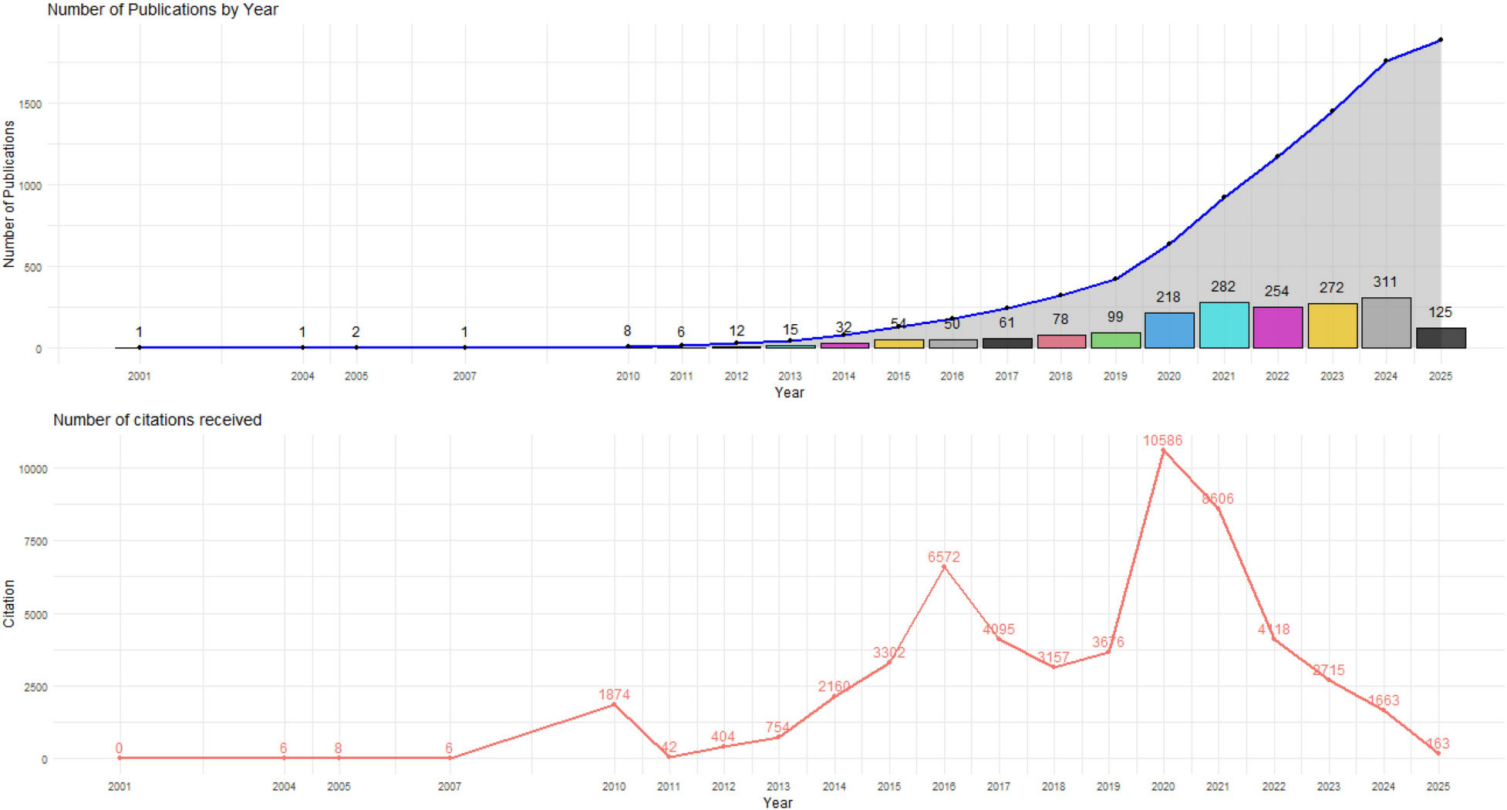
Temporal evolution of scientific production and citation impact in digital epidemiology and public health surveillance (2001–2025).

Figure 2 shows the temporal evolution of scientific production and impact measured in citations within the field of digital epidemiology and public health surveillance between 2001 and 2025. The results evidence a sustained growth in annual publication productivity, accompanied by notable fluctuations in received citations, reflecting both the degree of field maturity and the influence of global health events.

Between 2001 and 2010, an incipient phase is observed, characterized by very low productivity (from one to eight annual publications) and a reduced number of citations. However, from 2010 onward, an inflection point is recorded with 1,874 citations, suggesting the consolidation of the first relevant studies on digital surveillance and real-time data use. The period 2014–2016 represents a stage of methodological and technological expansion, with a considerable increase in publications (from 32 to 50) and accelerated growth in citations, reaching a maximum of 6,572 in 2016. This behavior coincides with the integration of big data and machine learning in epidemiological monitoring, as well as the rise of digital health on the global agenda.

The greatest increase occurs between 2019 and 2021, when the number of articles rises from 99 to 282. This surge is closely linked to the COVID-19 pandemic, which drove the development of predictive models, social media analysis, and genomic surveillance, generating the peak in citations in 2020 (10,586). From 2022 onward, although production remains high (more than 250 articles per year), citations decline progressively, indicating a process of stabilization and thematic diversification post-health emergency.

This accelerated growth was likely driven by several converging factors. First, the COVID-19 pandemic generated an unprecedented need for real-time surveillance, predictive modeling, and rapid public health decision-making. Second, the emergency context intensified international collaboration and increased institutional investment in digital monitoring systems, data platforms, and AI-assisted analytics. Third, the wider availability of mobile technologies, social media data, and interoperable digital infrastructures expanded the feasibility of digital epidemiology across multiple settings. Together, these factors help explain why scientific production increased sharply during the pandemic period and remained high afterward.

Finally, the 2024 and 2025 data reflect a possible trend shift. In 2024, 311 publications are reached, the highest value of the period, but with a notable decline in citations (1,663). This suggests that, while digital epidemiology continues to be an active field, scientific attention may be shifting toward new research lines or the consolidation of normative and ethical frameworks. Overall, the figure shows a typical trajectory of scientific maturation: a prolonged exploratory phase, followed by an explosion of productivity driven by global crises, and subsequently a stage of theoretical consolidation and thematic diversification.

### 3.2 Most influential sources

Table 1 presents the most influential sources in the scientific production on digital epidemiology and public health surveillance during the period 2000–2025. The data evidence a strong concentration of publications in journals specialized in digital health, medical informatics, and epidemiological surveillance, reflecting the interdisciplinary nature of the field and its close relationship with computational sciences and public health.

**Table 1:** Most influential publication sources in digital epidemiology and public health surveillance (2000–2025).

Source.title	Quantity	Citations
Journal of Medical Internet Research	260	12,406
BMJ Open	83	1,162
JMIR Public Health and Surveillance	68	2,639
JMIR Infodemiology	40	312
Yearbook of Medical Informatics	37	799
JMIR Formative Research	37	225
BMJ Health and Care Informatics	23	310
International Journal of Environmental Research and Public Health	20	1,102
IEEE Access	20	638

The *Journal of Medical Internet Research* positions itself as the primary dissemination source, with 260 articles and a total of 12,406 citations. This result confirms its role as a central hub in the dissemination of research on digital health, telemedicine, and data-based epidemiology, establishing itself as the main reference at the intersection of technology and health. It is followed by *BMJ Open* with 83 articles and 1,162 citations, and *JMIR Public Health and Surveillance* with 68 publications and 2,639 citations, both oriented toward the application of digital tools and surveillance methodologies in public health settings.

*JMIR Infodemiology*, with 40 articles and 312 citations, emerges as a recently created publication focused on the analysis of information flows, misinformation, and digital behaviors during health crises, evidencing the expansion of the subdiscipline known as ‘infodemiology’.

Other relevant journals, such as *Yearbook of Medical Informatics* (37 articles, 799 citations) and *JMIR Formative Research* (37 articles, 225 citations), contribute evidence of sustained interest in emerging technologies and the formative phase of digital health projects. For its part, *BMJ Health and Care Informatics* (23 articles, 310 citations) and the *International Journal of Environmental Research and Public Health* (20 articles, 1,102 citations) reflect the integration of medical informatics and environmental approaches in global health surveillance. Finally, *IEEE Access* (20 articles, 638 citations) stands out as the main non-strictly biomedical technological publication channel, indicating the contribution of engineering and data science to strengthening digital surveillance.

Collectively, the table reveals an editorial ecosystem dominated by open-access journals with high international visibility, which has favored the rapid dissemination of knowledge in a field characterized by its dynamism, interdisciplinarity, and social relevance in the face of global public health challenges.

### 3.3 Institutional collaboration networks

**Figure 3:**
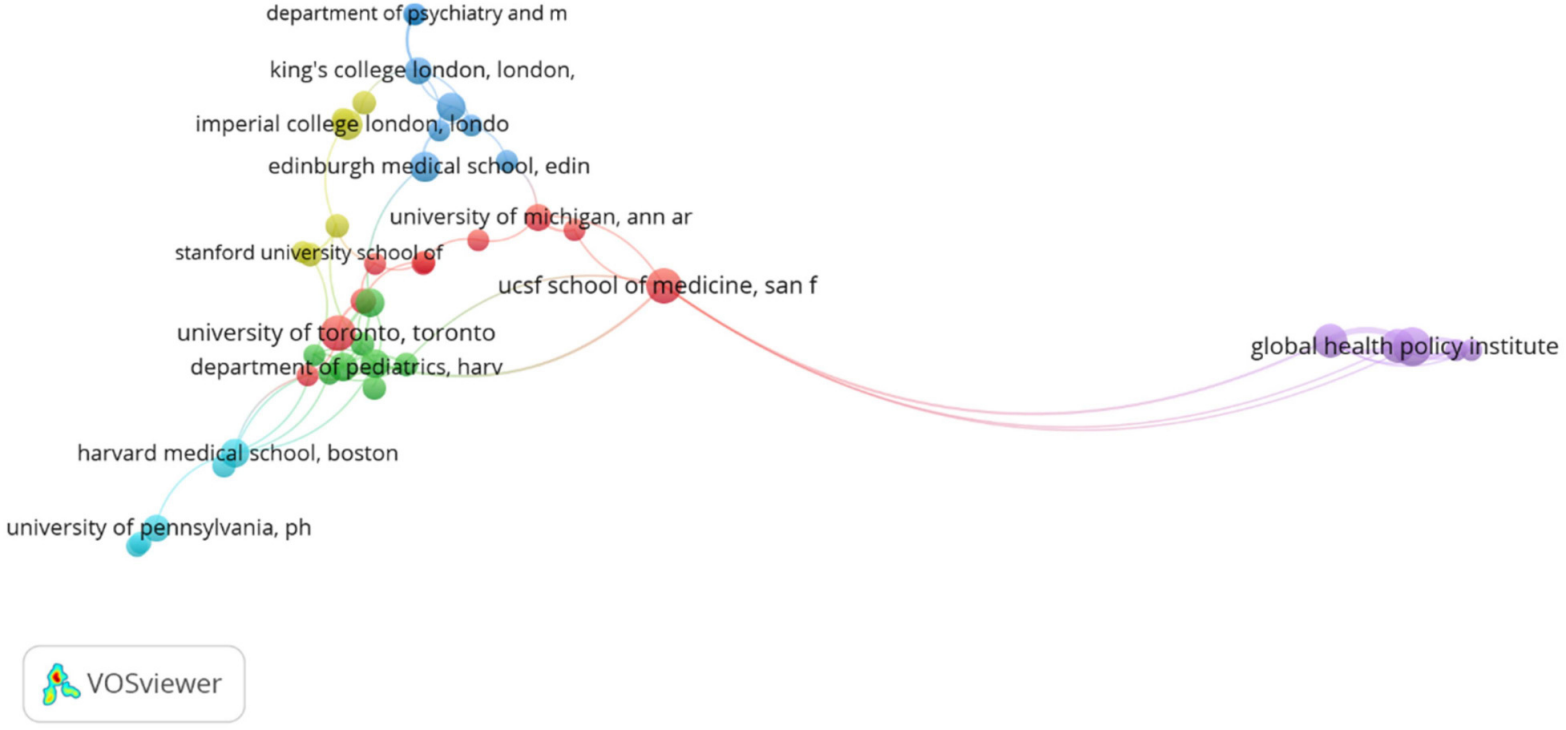
Institutional collaboration network in digital epidemiology and public health surveillance.

Figure 3 represents the institutional collaboration network in the field of digital epidemiology and public health surveillance, evidencing the formation of scientific cooperation clusters among prestigious academic institutions and research centers internationally. The nodes reflect the volume of publications, while the links and line thickness indicate the intensity of collaborative relationships, measured through total link strength.

The analysis shows a highly concentrated structure in institutions from the United States and Europe, with significant connectivity among elite universities. At the core of the network are Harvard Medical School (Boston), the University of Pennsylvania (Philadelphia), the University of Toronto Faculty of Medicine (Canada), Stanford University School of Medicine, and the UCSF School of Medicine (San Francisco). These entities form a dense cluster characterized by strong interconnections, reflecting cooperation between departments of medicine, biomedical informatics, and public health for the development of technologies applied to health surveillance.

The Harvard T.H. Chan School of Public Health and the Department of Pediatrics of the same university stand out for their articulating role between North American and European institutions. In the European context, King's College London, Imperial College London, and Edinburgh Medical School excel, establishing links with North American centers such as Stanford and Michigan. This transatlantic co-authorship pattern suggests a sustained flow of knowledge between biomedical and technological networks oriented toward the analysis of epidemiological data.

The Global Health Policy Institute in San Diego appears as a peripheral node but with high link strength (29), indicating its function as a convergence center in interdisciplinary projects on global health policy. Its direct connection to the UCSF School of Medicine and the University of Michigan reflects an orientation toward studies on governance, international cooperation, and the application of digital epidemiology in public policies.

Other relevant actors, though with lower relational density, include Boston Children's Hospital (463 citations, link strength 15), the Institute of Health Policy, Management and Evaluation in Toronto (633 citations, strength 16), and the University of Waterloo (126 citations, strength 15). These centers, along with the University of Texas System (strength 17), consolidate an academic network that combines clinical research, applied informatics, and health data management.

Collectively, the institutional network evidences collaboration predominantly concentrated in high-income countries, with central nodes in North America and Western Europe. This configuration suggests the existence of a mature and cooperative scientific structure, where knowledge production on digital epidemiology is sustained by high-specialization inter-university alliances and interactions between medical, technological, and public policy institutions.

### 3.4 International collaboration networks

**Figure 4:**
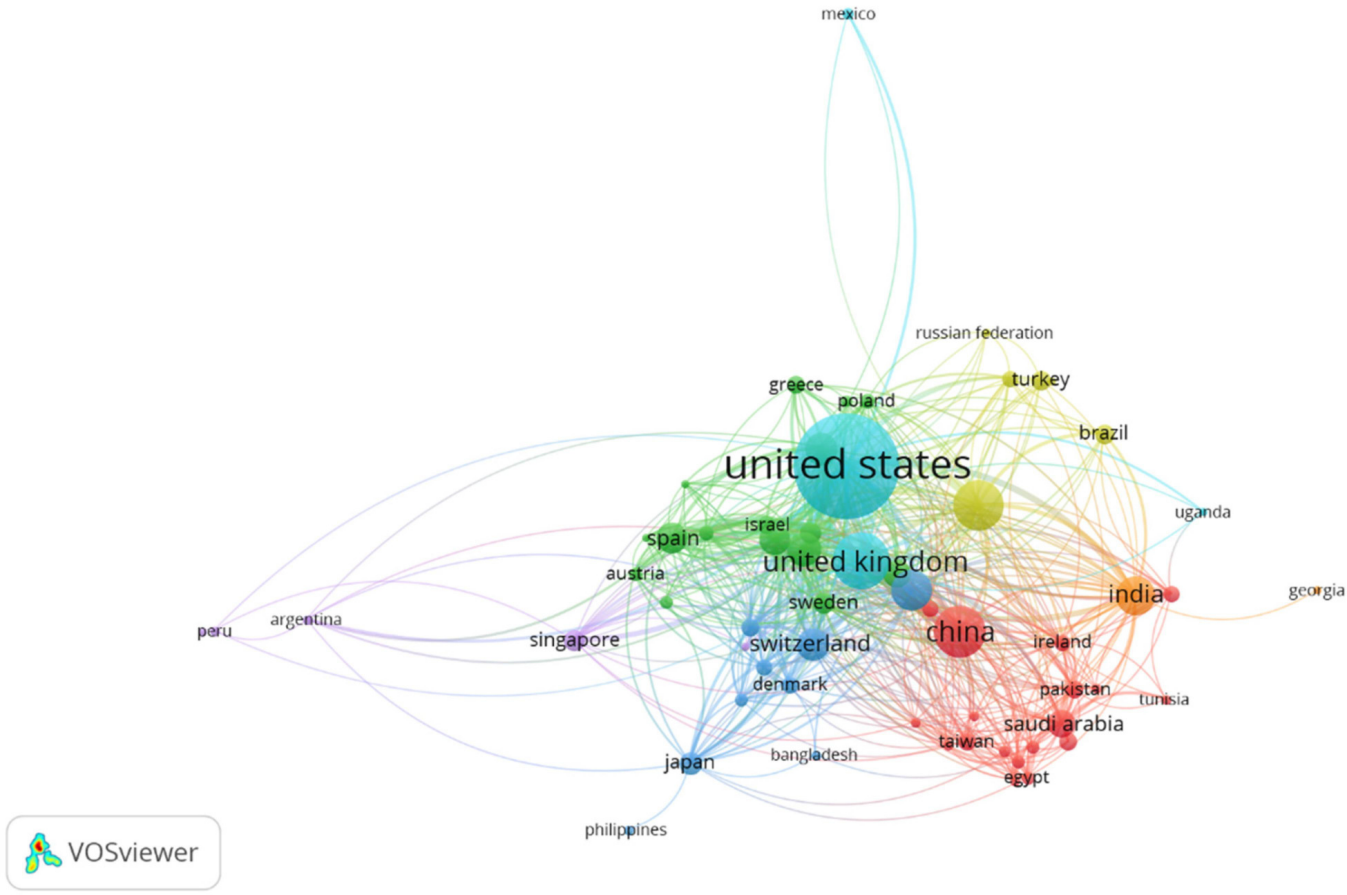
International collaboration network in digital epidemiology and public health surveillance.

Figure 4 represents the international collaboration network in the field of digital epidemiology and public health surveillance between 2000 and 2025. The nodes correspond to participating countries, with their size proportional to the number of published documents, while the density of links indicates the strength of collaborative relationships (total link strength) between nations.

The map reveals a global structure dominated by high-income countries, particularly the United States, United Kingdom, China, Germany, and Switzerland, which form the main core of scientific cooperation. The United States positions itself as the most influential actor in the system, with 668 publications, an average of 34 citations, and a total link strength of 382, evidencing its central role in knowledge production and articulation on digital surveillance and public health. Its position at the center of the network and the large number of connections with European and Asian countries reflects its leadership function and role as a bridge between different research poles.

The United Kingdom (198 documents, link strength 273) ranks second in influence, acting as an intermediation node between Europe, America, and Asia. It is followed by China (163 publications, link strength 106) and India (90, link strength 89), both emerging as poles of scientific production with growing prominence in the development of artificial intelligence and big data analysis applied to health surveillance. These countries form an Asian subcluster characterized by strong regional interconnection and a tendency to establish links with North American and European centers.

In continental Europe, Germany (69 publications, link strength 149), Italy (64, link strength 99), Spain (56, link strength 100), and Switzerland (62, link strength 126) stand out, countries that consolidate a dense network of transnational cooperation oriented toward technological innovation, genomic surveillance, and ethical data management in health. Belgium, with 2,894 citations, excels for its high scientific visibility despite its lower publication volume, suggesting high-impact production.

Beyond this Euro-North American axis, significant participation is observed from Australia (97 publications, link strength 92) and Canada (149, link strength 150), both acting as bridges between Anglophone research systems. In the Southern Hemisphere, Brazil (24 publications, link strength 21) and Mexico (9 publications, 1,107 citations) represent the main Latin American foci, although their connection to the global core remains limited.

The analysis also evidences the growing incorporation of middle-income countries, particularly from the Middle East and South Asia, such as Saudi Arabia (link strength 60), Pakistan (32), and Egypt (28), which strengthen South-South cooperation on infectious disease surveillance and the use of mobile health technologies.

Collectively, the global network shows a clear asymmetry in scientific production and collaboration, concentrated in Northern countries, but with signs of geographic diversification and openness toward emerging regions. This pattern suggests that digital epidemiology is consolidating as an internationally interconnected field, where cooperation between countries with advanced technological capabilities and those in development is fundamental to addressing global public health challenges and digital epidemiological surveillance.

### 3.5 Thematic area

**Figure 5:**
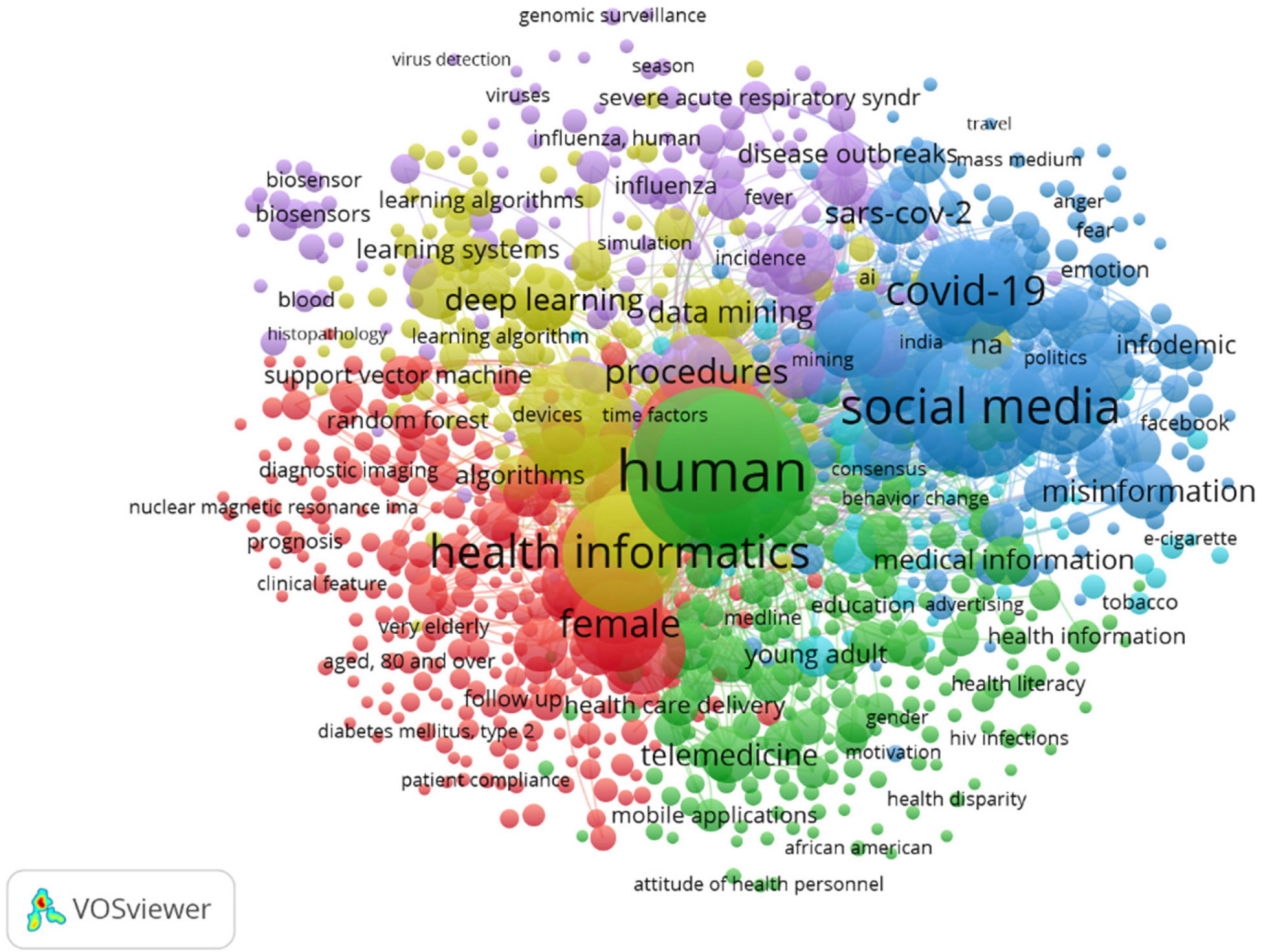
Keyword Co-occurrence network in digital epidemiology and public health surveillance (2000–2025).

Figure 5 represents the keyword co-occurrence map in the literature on digital epidemiology and public health surveillance. Each node corresponds to a key term, its size reflects the frequency of appearance (occurrences), and the proximity between nodes indicates thematic association (total link strength). The analysis reveals five macro-conceptual fields that structure the field: (i) epidemiological surveillance and digital public health, (ii) social platforms and infodemiology, (iii) artificial intelligence and data analytics, (iv) telemedicine and connected health, and (v) response to large-scale health emergencies, particularly the COVID-19 pandemic.

First, a biomedical and clinical core is observed, articulated around terms such as *human* (939 occurrences, link strength 18,874), *female* (285 occurrences, 6,979), *male* (269 occurrences, 6,618), *adult* (259 occurrences, 6,381), and *aged* (100 occurrences, 2,661). This cluster reflects that much of the literature has a population-based and clinical foundation, with studies analyzing outcomes in different age groups (*young adult*, 58 occurrences; *middle aged*, 104 occurrences; *very elderly*, 24 occurrences) and risk profiles. Words like *comorbidity* (21 occurrences, strength 599), *cardiovascular disease* (19 occurrences, 484), and *chronic disease* (33 occurrences, 713) suggest that digital epidemiology is not limited to infectious diseases but also applies to the tracking of chronic conditions and longitudinal clinical management, for example, in *diabetes mellitus* (26 occurrences, 643) and *hypertension* (17 occurrences, 385).

Second, an axis strongly linked to digital public health and information management is identified, centered on terms such as *health informatics* (465 occurrences, 6,042), *public health* (312 occurrences, 5,537), *public health surveillance* (160 occurrences, 2,481), *disease surveillance* (59 occurrences, 1,406), and *epidemiology* (162 occurrences, 3,541). This block incorporates both technical components (*electronic health record*, 79 occurrences, 1,600; *information processing*, 79 occurrences, 1,835) and governance components (*privacy*, 41 occurrences, 599; *health policy*, 22 occurrences, 418; *health equity*, 20 occurrences, 310). The presence of terms like *risk assessment* (52 occurrences, 1,232), *prediction* (60 occurrences, 1,578), and *forecasting* (52 occurrences, 942) evidences the emphasis on predictive surveillance and early threat evaluation (49).

Third, the figure shows a technological cluster associated with artificial intelligence and data science. Concepts such as *machine learning* (453 occurrences, 6,928), *artificial intelligence* (290 occurrences, 3,926), *data mining* (109 occurrences, 1,738), *deep learning* (134 occurrences, 1,795), *support vector machine* (52 occurrences, 1,176), *random forest* (40 occurrences, 957), and *natural language processing* (158 occurrences, 2,755) have high frequency and high link values, indicating that advanced computational analytics has been fully integrated into epidemiological surveillance (42). Words like *classification* (41 occurrences, strength 503), *diagnostic accuracy* (25 occurrences, 573), and *predictive value* (32 occurrences, 917) point to concrete applications in assisted diagnosis, risk stratification, and clinical decision-making. This same block includes concepts like *genomic surveillance* (10 occurrences, 119) and *whole genome sequencing* (14 occurrences, 338), linking digital surveillance with genomic surveillance strategies and viral variant tracking.

Fourth, a subfield centered on digital platforms, social networks, and infodemiology is observed, with terms such as *social media* (623 occurrences, 9,822), *misinformation* (112 occurrences, 1,908), *infodemic* (70 occurrences, 1,450), *infodemiology* (367 occurrences, 6,011), *twitter* (173 occurrences, 2,715), *sentiment analysis* (67 occurrences, 1,222), and *information dissemination* (49 occurrences, 961). This cluster describes the use of data from social networks to monitor behaviors, perceptions, and public narratives during epidemic outbreaks (44). The association between *social media* and terms like *vaccine hesitancy* (25 occurrences, 463), *public opinion* (45 occurrences, 1,070), and *trust* (26 occurrences, 600) indicates that digital surveillance is not only oriented toward biological or clinical indicators but also toward sociocultural dynamics and the acceptance of health interventions (43). The weight of *google trends* (38 occurrences, strength 599) confirms the role of infoveillance (*infoveillance*, 139 occurrences, 2,451) as an early warning tool (45).

A fifth thematic axis is associated with responses to health emergencies and global epidemic crises. Concepts such as *covid-19* (374 occurrences, 6,365), *sars-cov-2* (134 occurrences, 2,946), *pandemic* (182 occurrences, 4,211), *disease outbreaks* (65 occurrences, 1,537), *vaccination* (59 occurrences, 1,358), and *surveillance* (98 occurrences, 1,226) appear closely linked. This block encompasses not only clinical characterization (*symptom*, *fever*, *pneumonia*, *severity of illness index*) but also operational components of public health such as *contact tracing*, *quarantine*, and *population surveillance* (36 occurrences, 721). The emphasis on *vaccine hesitancy* and *misinformation* reflects that pandemic management integrated epidemiological surveillance with communicational surveillance and social risk analysis (46–48).

Finally, applied themes to digital health and remote care environments emerge, grouping terms such as *telemedicine* (108 occurrences, 1,988), *mhealth* (73 occurrences, 914), *mobile health* (74 occurrences, 930), *mobile applications* (37 occurrences, 669), *wearable sensors* (9 occurrences, 134), and *patient monitoring* (23 occurrences, 599). This subfield indicates the expansion of health surveillance toward distributed ecosystems, in which clinical and behavioral data are captured continuously through mobile devices, electronic records, and portable technologies (41). The connection between *telemedicine*, *health care delivery* (53 occurrences, 1,013), *health service* (24 occurrences, 608), and *patient safety* (13 occurrences, 298) suggests that the use of digital tools is no longer solely for population monitoring but also for direct support in clinical practice and continuity of care.

Collectively, Figure 4 shows that digital epidemiology has consolidated as a multidimensional field that articulates epidemiological surveillance, advanced algorithmic analytics, social dynamics of information, and technology-mediated clinical care. The map evidences that the field has evolved from traditional descriptive approaches toward real-time, highly computational surveillance frameworks sensitive to social behavior and directly integrated into care practice. This technological-health-social convergence is consistent with public health's shift toward predictive, personalized, and connected models.

## 4 Qualitative analysis

### 4.1 Descriptive summary of the studies

This section provides a panoramic view of the investigative landscape on *Digital Epidemiology and Public Health Surveillance: Scientometric Mapping of Emerging Technologies and Challenges*, emphasizing the integration of advanced digital technologies such as artificial intelligence (AI), the Internet of Things (IoT), big data analytics, and health informatics within epidemiological surveillance systems. The fifty most cited studies analyzed present a methodological convergence between bibliometric approaches, systematic reviews, and applied empirical studies, highlighting the use of AI-driven predictive models, real-time monitoring systems, and automated analysis of clinical and social data.

Collectively, the investigations demonstrate significant advances in outbreak prediction capacity, optimization of health resources, and improvement in data-based decision-making. However, persistent challenges related to data quality and interoperability, technological infrastructure, and specialized personnel training are identified. The most recurrent ethical considerations revolve around privacy, algorithmic transparency, and responsible governance of sensitive data use.

From an equity perspective, the studies evidence a geographic concentration in high-income countries—primarily the United States, United Kingdom, China, and Canada—which reflects gaps in technological access and inequalities in the implementation capacity of digital solutions. Nevertheless, initiatives emerge in middle-income regions seeking to adapt digital epidemiology to local contexts through mobile tools, telemedicine, and participatory surveillance.

In summary, this comparison reveals a trend toward the consolidation of a global digital public health ecosystem, where technological innovation coexists with ethical, structural, and social challenges. The integrated analysis of technological scope, effectiveness, equity, and implementation barriers contributes to delineating the opportunities and limitations that determine the evolution of epidemiological surveillance in the digital era.

### 4.2 Technological scope

The fifty most cited studies cover a broad spectrum of emerging technologies applied to epidemiological surveillance, including artificial intelligence (AI), machine learning, big data analytics, health informatics, social media mining, sensors and Internet of Things (IoT), natural language processing (NLP), and mobile health tools. Collectively, they reflect an interdisciplinary approach that integrates public health, informatics, and data science to strengthen surveillance and response systems to health emergencies (1, 7, 8).

AI and machine learning constitute the predominant technologies, used for outbreak prediction, early disease detection, and health resource optimization. These are frequently combined with IoT and big data to increase the capacity for massive data analysis and improve diagnostic precision (5, 9, 10, 42).

Various studies highlight the incorporation of non-conventional data sources—such as social networks, electronic health records, and mobile devices—to complement traditional epidemiological surveillance systems, enabling continuous monitoring and real-time response (2, 11, 12, 39, 41, 44).

### 4.3 Effectiveness measures

Thirty-five of the most cited studies report significant improvements in predictive and diagnostic performance, evidencing increases in precision, sensitivity, and specificity of AI-based models compared to conventional surveillance methods (1, 9, 13).

Other works demonstrate the practical utility of these technologies in early outbreak detection and continuous population monitoring, contributing to reduced response times and proactive planning in public health (3, 14, 15, 43).

Several studies acknowledge the still experimental nature of many technological applications, pointing to the need for additional validation, integration in real environments, and consolidation of interoperability frameworks between systems (16–18).

### 4.4 Ethical and privacy considerations

Forty studies explicitly address the ethical implications arising from the use of digital technologies in health, emphasizing the protection of privacy, responsible data management, and transparency in algorithmic models (4, 19, 20).

The most frequent concerns relate to data governance, informed consent, and the need for normative frameworks that regulate the secure exchange of information between institutions and countries (6, 12, 21).

Calls for interdisciplinary collaboration and the creation of solid ethical frameworks that ensure responsible and transparent use of artificial intelligence in public health are also repeatedly identified (5, 15, 18).

### 4.5 Equity and accessibility

Thirty studies analyze the equity and accessibility aspects linked to the adoption of digital technologies in epidemiological surveillance, noting that while technological innovations improve health response capacity, their implementation tends to concentrate in high-income countries and urban settings (13, 16).

In several studies, equity is mentioned implicitly, focused on the need to extend access to digital systems to vulnerable populations, rural communities, and groups with lower technological literacy (10, 22, 23).

The importance of social acceptance and the population impact of technological innovations is recognized, highlighting the role of digital education and citizen participation in reducing technological gaps (2, 19, 40, 50).

### 4.6 Implementation barriers

Forty-five studies identify common structural barriers that limit the effective adoption of digital technologies in public health, including deficiencies in technological infrastructure, insufficient interoperability, lack of data standardization, and shortage of specialized technical personnel (1, 24, 25).

The most recurrent technical barriers relate to the explainability of AI models, integration with existing health systems, and computational limitations, especially in resource-constrained contexts (7, 23, 26).

Finally, normative and regulatory challenges are highlighted, such as the absence of clear data governance policies, lack of unified ethical frameworks, and the need for international cooperation for the development of interoperable and sustainable systems (6, 8, 11).

## 5 Discussion

The findings derived from the analysis of the fifty most cited studies evidence that digital epidemiology has evolved toward an interdisciplinary ecosystem that combines artificial intelligence (AI), big data analytics, social media mining, the Internet of Things (IoT), and mobile health systems to strengthen global epidemiological surveillance (1, 7, 8). This technological convergence enables continuous monitoring of population data and early outbreak detection, improving the predictive capacity of traditional systems (2, 3, 14).

The predominance of AI and machine learning confirms their role as central tools for processing large volumes of health information and optimizing responses to emergencies. However, several studies underscore that these models require extensive validations in real scenarios and robust interoperability frameworks (5, 17, 18). The reported efficacy—manifested in diagnostic precision and reduced alert times—is countered by the lack of data flow standardization and unequal availability of digital infrastructure, especially in middle- and low-income countries (11, 16).

Beyond the reported benefits, the evidence reveals important methodological and practical limitations that require further scrutiny. While many studies report improvements in predictive accuracy, these findings are often based on controlled or retrospective datasets, limiting their generalizability to real-world public health contexts (16, 17).

Moreover, inconsistencies emerge regarding the scalability of digital epidemiology systems. While some studies highlight successful implementations in high-income settings (11), others point to failures related to data fragmentation, lack of interoperability, and insufficient institutional capacity (24, 25). This suggests that technological effectiveness is highly context-dependent.

Another critical issue concerns the reliability of alternative data sources such as social media. Although these sources enable real-time monitoring, they are also prone to bias, misinformation, and representativeness problems, which may compromise the validity of epidemiological inferences (2, 14).

Finally, the literature reveals a gap between methodological sophistication and practical implementation. Advanced AI models often lack explainability and transparency, limiting their adoption in decision-making environments where accountability is required (18, 26).

These findings suggest that the future of digital epidemiology depends not only on technological advancement but also on the development of robust validation frameworks, context-sensitive implementation strategies, and interdisciplinary governance mechanisms.

The ethical dimension emerges as a transversal constant. Forty of the reviewed studies identify the urgent need to strengthen data governance frameworks, ensure health information privacy, and mitigate algorithmic biases that can reproduce structural inequalities (4, 8, 20). In this sense, transparency, model explainability, and accountability in AI-assisted decision-making are positioned as indispensable ethical pillars (6, 12, 21).

Likewise, the studies reflect a persistent gap in digital accessibility and equity. Despite technological advances, most applications concentrate in urban or institutional contexts, while rural and vulnerable populations continue to face limitations in connectivity and digital literacy (13, 22, 23). This confirms that the digital transformation in public health does not depend solely on technological innovation but also on inclusive policies that promote local capacities and citizen participation.

The identified implementation barriers—infrastructure limitations, deficient interoperability, shortage of technical personnel, and lack of international standardization—coincide with obstacles reported in the digital health literature (24–26). Normative challenges are equally critical: the absence of unified global policies for ethical data management and reliance on fragmented regulatory frameworks hinder transnational integration of digital surveillance (6, 11, 18).

Collectively, the discussion evidences that digital epidemiology has transitioned from an experimental phase to a consolidated public health paradigm, but it still faces challenges in equity, governance, and empirical validation. The 50 most cited studies delineate a rapidly expanding scientific field, where the combination of AI, massive data, and alternative sources—such as social networks or wearable sensors—redefines the boundaries between surveillance, prediction, and health action. However, its sustainable impact will depend on the ethical, institutional, and social articulation that ensures transparency, public trust, and digital equity in health decision-making (1, 4, 6).

### 5.1 Integration with bibliometric findings

The bibliometric analysis complements and reinforces the described thematic patterns. Publication trends demonstrate exponential growth since 2010, with an inflection point during the 2020–2021 period, coinciding with the COVID-19 pandemic and the massive adoption of digital technologies in epidemiological surveillance. This increase in scientific production, accompanied by a proportional rise in citations, reflects not only academic interest but also institutional and social urgency to innovate in health monitoring mechanisms.

In terms of productivity, the *Journal of Medical Internet Research* positions itself as the main publication source, with over 10,000 accumulated citations, followed by *BMJ Open* and *JMIR Public Health and Surveillance*, evidencing editorial concentration in journals specialized in digital health and scientific communication. This concentration reinforces the field's maturity, where academic production converges in high-visibility spaces with editorial lines centered on technology and public health.

From a geographic perspective, the international collaboration analysis reveals clear leadership from the United States (668 publications, 34 average citations) and the United Kingdom (198 publications), followed by China, Canada, Germany, Italy, and Australia. These countries configure dense cooperation networks, acting as articulating nodes of global production. However, the limited contribution from Latin America, Africa, and Southeast Asia confirms the existence of a structural gap in research capacity and technological adoption, consistent with the equity and accessibility limitations identified in Table 2.

**Table 2:** Qualitative analysis of the 50 most cited studies: Key Dimensions in technological scope, effectiveness, ethics, equity, and Implementation barriers.

Study	Technological scope	Effectiveness measures	Ethical and privacy considerations	Equity and accessibility	Implementation barriers	Total citations
Mohanty et al. (1)	Artificial intelligence/machine learning; mobile health, sensors and IoT; digital epidemiological surveillance	Improvement in predictive/diagnostic performance (precision, sensitivity, specificity).	Recognizes the need for ethical frameworks and responsible management of digital health data.	Broad population focus, with attention to social impact and public acceptance.	Infrastructure, interoperability and integration with real systems; technical capabilities and personnel training.	3,420
Ravì et al. (7)	Health informatics/electronic health records; artificial intelligence/machine learning; mobile health, sensors and IoT	Early detection and rapid response to outbreaks or critical events.	Recognizes the need for ethical frameworks and responsible management of digital health data.	Broad population focus, with attention to social impact and public acceptance.	Limitations related to technological maturity and large-scale adoption.	1,574
Chew and Eysenbach (2)	Artificial intelligence/machine learning; social media mining; digital epidemiological surveillance	Early detection and rapid response to outbreaks or critical events.	Recognizes the need for ethical frameworks and responsible management of digital health data.	Broad population focus, with attention to social impact and public acceptance.	Limitations related to technological maturity and large-scale adoption.	1,265
Holzinger (5)	Big data and large-scale analytics; health informatics/electronic health records; artificial intelligence/machine learning	Reported advances in practical utility and potential application in public health.	Recognizes the need for ethical frameworks and responsible management of digital health data.	Equity and accessibility mentioned generally or implicitly.	Technical capabilities and personnel training.	732
Abd-alrazaq et al. (24)	Health informatics/electronic health records; artificial intelligence/machine learning; social media mining; digital epidemiological surveillance	Capacity for continuous monitoring and population/individual follow-up.	Recognizes the need for ethical frameworks and responsible management of digital health data.	Broad population focus, with attention to social impact and public acceptance.	Infrastructure, interoperability and integration with real systems; regulatory and governance challenges.	610
Andreu-Perez et al. (8)	Big data and large-scale analytics; health informatics/electronic health records; artificial intelligence/machine learning; mobile health, sensors and IoT	Reported advances in practical utility and potential application in public health.	Issues on privacy and data confidentiality, ethical governance of data and algorithm use.	Applies to specific clinical populations (chronic patients, older adults).	Regulatory and governance challenges.	602
Ahmad and Murad (3)	Artificial intelligence/machine learning; social media mining; digital epidemiological surveillance	Early detection and rapid response to outbreaks or critical events.	Recognizes the need for ethical frameworks and responsible management of digital health data.	Broad population focus, with attention to social impact and public acceptance.	Infrastructure, interoperability and integration with real systems.	492
Abd-Alrazaq et al. (27)	Health informatics/electronic health records; artificial intelligence/machine learning; social media mining; digital epidemiological surveillance	Capacity for continuous monitoring and population/individual follow-up.	Recognizes the need for ethical frameworks and responsible management of digital health data.	Broad population focus, with attention to social impact and public acceptance.	Infrastructure, interoperability and integration with real systems; regulatory and governance challenges.	468
Deng et al. (22)	Health informatics/electronic health records; mobile health, sensors and IoT	Reported advances in practical utility and potential application in public health.	Recognizes the need for ethical frameworks and responsible management of digital health data.	Applies to specific clinical populations (chronic patients, older adults).	Limitations related to technological maturity and large-scale adoption.	385
Boon-Itt et al. (11)	Health informatics/electronic health records; artificial intelligence/machine learning; social media mining; digital epidemiological surveillance	Improvement in predictive/diagnostic performance (precision, sensitivity, specificity).	Recognizes the need for ethical frameworks and responsible management of digital health data.	Broad population focus, with attention to social impact and public acceptance.	Limitations related to technological maturity and large-scale adoption.	365
Mavragani et al. (38)	Big data and large-scale analytics; health informatics/electronic health records; artificial intelligence/machine learning; digital epidemiological surveillance	Reported advances in practical utility and potential application in public health.	Recognizes the need for ethical frameworks and responsible management of digital health data.	Equity and accessibility mentioned generally or implicitly.	Limitations related to technological maturity and large-scale adoption.	337
Andreu-Perez et al. (19)	Health informatics/electronic health records; artificial intelligence/machine learning; social media mining; mobile health, sensors and IoT	Improvement in predictive/diagnostic performance (precision, sensitivity, specificity).	Recognizes the need for ethical frameworks and responsible management of digital health data.	Applies to specific clinical populations (chronic patients, older adults).	Infrastructure, interoperability and integration with real systems.	329
Ardabili et al. (9)	Health informatics/electronic health records; artificial intelligence/machine learning; digital epidemiological surveillance	Improvement in predictive/diagnostic performance (precision, sensitivity, specificity).	Recognizes the need for ethical frameworks and responsible management of digital health data.	Broad population focus, with attention to social impact and public acceptance.	Limitations related to technological maturity and large-scale adoption.	324
Bragazzi et al. (4)	Big data and large-scale analytics; artificial intelligence/machine learning; digital epidemiological surveillance	Early detection and rapid response to outbreaks or critical events.	Issues on ethical governance of data and algorithm use.	Broad population focus, with attention to social impact and public acceptance.	Limitations related to technological maturity and large-scale adoption.	318
Chowdhury et al. (10)	Artificial intelligence/machine learning; digital epidemiological surveillance	Improvement in predictive/diagnostic performance (precision, sensitivity, specificity).	Recognizes the need for ethical frameworks and responsible management of digital health data.	Equity and accessibility mentioned generally or implicitly.	Limitations related to technological maturity and large-scale adoption.	307
Fagherazzi et al. (12)	Social media mining; digital epidemiological surveillance	Capacity for continuous monitoring and population/individual follow-up.	Issues on ethical governance of data and algorithm use.	Broad population focus, with attention to social impact and public acceptance.	Infrastructure, interoperability and integration with real systems; regulatory and governance challenges.	303
Gündüz (28)	Health informatics/electronic health records; artificial intelligence/machine learning	Improvement in predictive/diagnostic performance (precision, sensitivity, specificity).	Recognizes the need for ethical frameworks and responsible management of digital health data.	Applies to specific clinical populations (chronic patients, older adults).	Limitations related to technological maturity and large-scale adoption.	292
Liu et al. (29)	Artificial intelligence/machine learning; mobile health, sensors and IoT; digital epidemiological surveillance	Improvement in predictive/diagnostic performance (precision, sensitivity, specificity).	Recognizes the need for ethical frameworks and responsible management of digital health data.	Equity and accessibility mentioned generally or implicitly.	Limitations related to technological maturity and large-scale adoption.	290
Miller et al. (30)	Health informatics/electronic health records; artificial intelligence/machine learning; mobile health, sensors and IoT	Reported advances in practical utility and potential application in public health.	Issues on transparency and explainability of models.	Applies to specific clinical populations (chronic patients, older adults).	Infrastructure, interoperability and integration with real systems.	289
Xue et al. (14)	Artificial intelligence/machine learning; social media mining; digital epidemiological surveillance	Early detection and rapid response to outbreaks or critical events.	Recognizes the need for ethical frameworks and responsible management of digital health data.	Broad population focus, with attention to social impact and public acceptance.	Limitations related to technological maturity and large-scale adoption.	285
Prosperi et al. (26)	Big data and large-scale analytics; health informatics/electronic health records; artificial intelligence/machine learning	Improvement in predictive/diagnostic performance (precision, sensitivity, specificity).	Issues on algorithmic bias and equity.	Broad population focus, with attention to social impact and public acceptance.	Limitations related to technological maturity and large-scale adoption.	281
Ristevski and Chen (17)	Big data and large-scale analytics; health informatics/electronic health records	Improvement in predictive/diagnostic performance (precision, sensitivity, specificity).	Issues on privacy and data confidentiality.	Equity and accessibility mentioned generally or implicitly.	Infrastructure, interoperability and integration with real systems.	277
Brewer et al. (13)	Health informatics/electronic health records; artificial intelligence/machine learning; mobile health, sensors and IoT	Early detection and rapid response to outbreaks or critical events.	Issues on algorithmic bias and equity.	Broad population focus, with attention to social impact and public acceptance.	Limitations related to technological maturity and large-scale adoption.	272
Lyu et al. (31)	Artificial intelligence/machine learning; social media mining; digital epidemiological surveillance	Reported advances in practical utility and potential application in public health.	Recognizes the need for ethical frameworks and responsible management of digital health data.	Broad population focus, with attention to social impact and public acceptance.	Limitations related to technological maturity and large-scale adoption.	266
Myslín et al. (32)	Artificial intelligence/machine learning; social media mining; digital epidemiological surveillance	Early detection and rapid response to outbreaks or critical events.	Recognizes the need for ethical frameworks and responsible management of digital health data.	Broad population focus, with attention to social impact and public acceptance.	Data quality and availability.	262
Low et al. (33)	Artificial intelligence/machine learning; social media mining; digital epidemiological surveillance	Improvement in predictive/diagnostic performance (precision, sensitivity, specificity).	Recognizes the need for ethical frameworks and responsible management of digital health data.	Equity and accessibility mentioned generally or implicitly.	Limitations related to technological maturity and large-scale adoption.	259
Herland et al. (34)	Big data and large-scale analytics; health informatics/electronic health records; artificial intelligence/machine learning; social media mining; digital epidemiological surveillance	Reported advances in practical utility and potential application in public health.	Recognizes the need for ethical frameworks and responsible management of digital health data.	Applies to specific clinical populations (chronic patients, older adults).	Limitations related to technological maturity and large-scale adoption.	256
Wu et al. (35)	Big data and large-scale analytics; health informatics/electronic health records; artificial intelligence/machine learning	Improvement in predictive/diagnostic performance (precision, sensitivity, specificity).	Recognizes the need for ethical frameworks and responsible management of digital health data.	Equity and accessibility mentioned generally or implicitly.	Limitations related to technological maturity and large-scale adoption.	256
Dadaczynski et al. (36)	Artificial intelligence/machine learning; social media mining; digital epidemiological surveillance	Early detection and rapid response to outbreaks or critical events.	Recognizes the need for ethical frameworks and responsible management of digital health data.	Broad population focus, with attention to social impact and public acceptance.	Limitations related to technological maturity and large-scale adoption.	252
Pastorino et al. (37)	Big data and large-scale analytics; artificial intelligence/machine learning; digital epidemiological surveillance	Improvement in predictive/diagnostic performance (precision, sensitivity, specificity).	Recognizes the need for ethical frameworks and responsible management of digital health data.	Broad population focus, with attention to social impact and public acceptance.	Infrastructure, interoperability and integration with real systems…(truncated in original)	219

The relationship between document quantity and citations also evidences the consolidation of specialized knowledge poles. For example, Canada, Germany, and Switzerland show high total link strength, suggesting a key role in the interconnection of multinational digital surveillance projects. In contrast, countries like Brazil, Mexico, Egypt, and the Czech Republic stand out for high average citation, attributable to pioneering studies with methodological or applied impact.

Collectively, the bibliometric indicators confirm that digital epidemiology configures as an expanding domain, but still concentrated in the global North, where resources, data infrastructure, and coordinated scientific agendas converge. This raises the need to promote interregional cooperation policies, open data exchange, and institutional strengthening in developing countries, to democratize access to technological innovations and reduce knowledge asymmetries.

The contrast between bibliometric results and the findings from the 50 most cited studies suggests that digital epidemiology is not only a scientific trend but a new governance model in public health. Data science applied to health has demonstrated its potential to transform epidemiological surveillance, but its sustainability will depend on how ethics, equity, and international cooperation are integrated into health systems.

The gathered evidence allows concluding that, while digital tools have demonstrated technical efficacy, their real impact requires accompaniment by a normative and social infrastructure that ensures responsible, transparent, and equitable data use. Thus, digital epidemiology should not be understood solely as a technological revolution but as a transition process toward a more connected, inclusive, and evidence-based public health.

## 6 Conclusions

The analysis of the fifty most cited studies, complemented by global bibliometric indicators, demonstrates that digital epidemiology is consolidating as an emerging and transformative field within contemporary public health. Its development is characterized by the convergence of technologies such as artificial intelligence (AI), big data, social media mining, the Internet of Things (IoT), and mobile health systems, which have expanded the predictive, diagnostic, and operational capacity of health surveillance systems (1, 7, 8).

However, technological advancement has not been homogeneous. The evidence points to a geographic and structural imbalance, where Northern countries concentrate most scientific production, digital infrastructure, and specialized human resources, while Latin American, African, and Southeast Asian contexts present lower visibility and participation. This pattern, already identified in the co-authorship and international collaboration analysis, underscores the urgency of promoting a more equitable, inclusive, and common-good-oriented science.

In summary, digital epidemiology has transitioned from an exploratory field to a consolidated global public health paradigm, in which predictive precision and response speed are integrated with principles of ethics, governance, and equity as indispensable components for its sustainability.

### 6.1 Practical implications

The results of this study offer important implications for public policy, health management, and professional practice.

- For public health systems, the findings suggest that the integration of emerging technologies can significantly improve early outbreak detection, resource allocation, and strategic planning. AI and big data, applied with ethical criteria and transparency, can optimize decision-making in health uncertainty scenarios (4, 6).
- At the institutional level, interoperability between systems, data standardization, and technical training of health personnel are necessary conditions for the sustainable adoption of digital tools.
- In the academic and training realm, competencies in data science, bioethics, and digital governance must be incorporated into public health curricula to prepare professionals capable of operating in technologically complex environments.
- Finally, international agencies and health ministries must strengthen global cooperation through open data exchange policies, harmonized regulatory frameworks, and funding oriented toward reducing the digital divide between regions.

### 6.2 Future research areas

Based on the analysis conducted, several priority lines for future research are identified:

- Evaluation of the real impact of digital technologies on population outcomes, beyond technical precision or detection metrics. It is necessary to measure their effect on morbidity reduction, health equity, and social trust.
- Development of ethical and algorithmic governance frameworks that ensure responsible AI use in epidemiological surveillance, with special emphasis on transparency, explainability, and personal data protection (18, 20).
- Comparative interregional studies on technological adoption, infrastructure, and institutional capacities, oriented toward identifying best implementation practices in resource-limited contexts.
- Integration of multidisciplinary approaches that combine public health, engineering, social sciences, and digital law to address the ethical and social challenges of automated surveillance.
- Application of longitudinal bibliometric and scientometric methods to monitor the thematic and collaborative evolution of the field, evaluating its degree of maturity and diversification.

## 7 Limitations

This study presents some limitations that should be considered when interpreting the results:

1. Dependence on the Scopus and Web of Science databases, which may have excluded relevant publications indexed in regional repositories or open-access sources not included in those databases.
2. Focus on the most cited studies, which, while reflecting the core of scientific influence, may bias understanding toward research with greater visibility, leaving out recent or emerging contributions.
3. Cross-sectional analysis, which does not allow capturing the temporal evolution of international collaboration or the delayed impact of technological innovations on health practice.
4. Methodological limitations in bibliometric indicators, such as variability in citation strategies or overrepresentation of Anglophone authors and institutions.

Despite these limitations, the results offer a comprehensive and updated view of the scientific landscape on digital epidemiology and public health surveillance, consolidating a solid foundation for future research that delves deeper into the intersection between technology, ethics, and health equity.

## References

[1] Mohanty SP Hughes DP Salathé M. Using deep learning for image-based plant disease detection. Front Plant Sci. (2016) 7:1419. 10.3389/fpls.2016.01419 27713752 PMC5032846

[2] Chew C Eysenbach G. Pandemics in the age of twitter: content analysis of tweets during the 2009 H1N1 outbreak. PLoS One. (2010) 5(11):e14118. 10.1371/journal.pone.0014118 21124761 PMC2993925

[3] Ahmad AR Murad HR. The impact of social media on panic during the COVID-19 pandemic in Iraqi Kurdistan: online questionnaire study. J Med Internet Res. (2020) 22(5):e19556. 10.2196/19556 32369026 PMC7238863

[4] Bragazzi NL Dai H Damiani G Behzadifar M Martini M Wu J. How big data and artificial intelligence can help better manage the COVID-19 pandemic. Int J Environ Res Public Health. (2020) 17(9):3176. 10.3390/ijerph17093176 32370204 PMC7246824

[5] Holzinger A. Interactive machine learning for health informatics: when do we need the human-in-the-loop? Brain Inform. (2016) 3:119–31. 10.1007/s40708-016-0042-6 27747607 PMC4883171

[6] Ronquillo CE Peltonen L-M Pruinelli L Chu CH Bakken S Beduschi A. Artificial intelligence in nursing: priorities and opportunities from an international invitational think-tank of the nursing and artificial intelligence leadership collaborative. J Adv Nurs. (2021) 77:3707–17. 10.1111/jan.14855 34003504 PMC7612744

[7] Ravì D Wong C Deligianni F Berthelot M Andreu-Perez J Lo B. Deep learning for health informatics. IEEE J Biomed Health Inform. (2017) 21(1):4–21. 10.1109/JBHI.2016.2636665 28055930

[8] Andreu-Perez J Poon CCY Merrifield RD Wong STC Yang G-Z. Big data for health. IEEE J Biomed Health Inform. (2015) 19(4):1193–208. 10.1109/JBHI.2015.2450362 26173222

[9] Ardabili SF Mosavi A Ghamisi P Filip F V´arkonyi-Ḱoczy AR Reuter U. COVID-19 outbreak prediction with machine learning. Algorithms. (2020) 13(10):249. 10.3390/a13100249

[10] Chowdhury MEH Rahman T Khandakar A Ayari MA Khan AU Khan MS. Automatic and reliable leaf disease detection using deep learning techniques. AgriEngineering. (2021) 3(2):294–312. 10.3390/agriengineering3020020

[11] Boon-Itt S Skunkan Y. Public perception of the COVID-19 pandemic on twitter: sentiment analysis and topic modeling study. JMIR Public Health Surveill. (2020) 6(4):e21978. 10.2196/21978 33108310 PMC7661106

[12] Fagherazzi G Goetzinger C Rashid MA Aguayo GA Huiart L. Digital health strategies to fight COVID-19 worldwide: challenges, recommendations, and a call for papers. J Med Internet Res. (2020) 22(6):e19284. 10.2196/19284 32501804 PMC7298971

[13] Brewer LC Fortuna KL Jones C Walker R Hayes SN Patten CA. Back to the future: achieving health equity through health informatics and digital health. JMIR Mhealth Uhealth. (2020) 8(1):e14512. 10.2196/14512 31934874 PMC6996775

[14] Xue J Chen J Hu R Chen C Zheng C Su Y. Twitter discussions and emotions about the COVID-19 pandemic: machine learning approach. J Med Internet Res. (2020) 22(11):e20550. 10.2196/20550 33119535 PMC7690968

[15] Valdez D Ten Thij M Bathina K Rutter LA Bollen J. Social media insights into US mental health during the COVID-19 pandemic: longitudinal analysis of twitter data. J Med Internet Res. (2020) 22(12):e21418. 10.2196/21418 33284783 PMC7744146

[16] Mavragani A Ochoa G. Google trends in infodemiology and infoveillance: methodology framework. JMIR Public Health Surveill. (2019) 5(2):e13439. 10.2196/13439 31144671 PMC6660120

[17] Ristevski B Chen M. Big data analytics in medicine and healthcare. J Integr Bioinform. (2018) 15(3):20170030. 10.1515/jib-2017-0030 29746254 PMC6340124

[18] Sounderajah V Ashrafian H Golub RM Shetty S de Fauw J Hooft L. Developing a reporting guideline for artificial intelligence-centred diagnostic test accuracy studies: the stard-AI protocol. BMJ Open. (2021) 11:e047709. 10.1136/bmjopen-2020-047709 34183345 PMC8240576

[19] Andreu-Perez J Leff DR Ip HMD Yang G-Z. From wearable sensors to smart implants-toward pervasive and personalized healthcare. IEEE Trans Biomed Eng. (2015) 62(12):2750–62. 10.1109/TBME.2015.2422751 25879838

[20] Saura JR Ribeiro-Soriano D Palacios-Marqués D. Assessing behavioral data science privacy issues in government artificial intelligence deployment. Gov Inf Q. (2022) 39(4):101679. 10.1016/j.giq.2022.101679

[21] Park HW Park S Chong M. Conversations and medical news frames on Twitter: infodemiological study on COVID-19 in South Korea. J Med Internet Res. (2020) 22(5):e18897. 10.2196/18897 32325426 PMC7202309

[22] Deng Z Mo X Liu S. Comparison of the middle-aged and older users’ adoption of mobile health services in China. Int J Med Inf. (2014) 83(3):210–24. 10.1016/j.ijmedinf.2013.12.002 24388129

[23] Hui N Sun X Niu S Luo X. Pegylated polyaniline nanofibers: antifouling and conducting biomaterial for electrochemical DNA sensing. ACS Applied Materials and Interfaces. (2017) 9(3):2914–23. 10.1021/acsami.6b11682 28026927

[24] Abd-alrazaq A Alhuwail D Househ M Hai M Shah Z. Top concerns of tweeters during the COVID-19 pandemic: a surveillance study. J Med Internet Res. (2020) 22(4):e19016. 10.2196/19016 32287039 PMC7175788

[25] Magrabi F Ammenwerth E Brender JB de Keizer NF Hyppönen H Nykänen P. Artificial intelligence in clinical decision support: challenges for evaluating AI and practical implications a position paper from the IMIA technology assessment & quality development in health informatics working group and the EFMI working group for assessment of health information systems. Yearb Med Inform. (2019) 28(1):128–34. 10.1055/s-0039-1677903 31022752 PMC6697499

[26] Prosperi M Guo Y Sperrin M Koopman JS Min JS He X. Causal inference and counterfactual prediction in machine learning for actionable healthcare. Nat Machine Intell. (2020) 2:369–75. 10.1038/s42256-020-0197-y

[27] Abd-Alrazaq A Alhuwail D Househ M Hamdi M Shah Z. Top concerns of tweeters during the COVID-19 pandemic: infoveillance study. J Med Internet Res. (2020) 22(4):e19016. 10.2196/19016 32287039 PMC7175788

[28] Gündüz H. Deep learning-based Parkinson’s disease classification using vocal feature sets. IEEE Access. (2019) 7:115540–51. 10.1109/ACCESS.2019.2936564

[29] Liu Y Tuleouva N Ramankulov E Revzin A. Aptamer-based electrochemical biosensor for interferon gamma detection. Anal Chem. (2010) 82(19):8131–6. 10.1021/ac101409t 20815336 PMC2948235

[30] Miller AS Cafazzo JA Seto E. A game plan: gamification design principles in mhealth applications for chronic disease management. Health Informatics J. (2016). 10.1177/1460458214537511 24986104

[31] Lyu JC Han EL Luli GK. COVID-19 vaccine-related discussion on twitter: topic modeling and sentiment analysis. J Med Internet Res. (2021) 23(6):e24435. 10.2196/24435 34115608 PMC8244724

[32] Myslín M Zhu S-H Chapman WW Conway M. Using Twitter to examine smoking behavior and perceptions of emerging tobacco products. J Med Internet Res. (2013) 15(8):e174. 10.2196/jmir.2534 23989137 PMC3758063

[33] Low DM Rumker L Talkar T Torous J Cecchi G Ghosh SS. Natural language processing reveals vulnerable mental health support groups and heightened health anxiety on Reddit during COVID-19: observational study. J Med Internet Res. (2020) 22(10):e22635. 10.2196/22635 32936777 PMC7575341

[34] Herland M Khoshgoftaar TM Wald R. A review of data mining using big data in health informatics. J Big Data. (2014) 1:2. 10.1186/2196-1115-1-2

[35] Wu P-Y Cheng C-W Kaddi CD Venugopalan J Hoffman R Wang MD. -Omic and electronic health record big data analytics for precision medicine. IEEE Trans Biomed Eng. (2017) 64(2):263–73. 10.1109/TBME.2016.2573285 27740470 PMC5859562

[36] Dadaczynski K Okan O Messer M Leung AYM Rosário R Darlington E. Digital health literacy and web-based information-seeking behaviors of university students in Germany during the COVID-19 pandemic: cross-sectional survey study. J Med Internet Res. (2021) 23(1):e24097. 10.2196/24097 33395396 PMC7813561

[37] Pastorino R Vito C Migliara G Glocker K Binenbaum I Ricciardi W. Benefits and challenges of big data in healthcare: an overview of the European initiatives. Eur J Public Health. (2019) 29(Supplement_3):23–7. 10.1093/eurpub/ckz168 31738444 PMC6859509

[38] Mavragani A Ochoa G Tsagarakis KP. Assessing the methods, tools, and statistical approaches in google trends research: systematic review. J Med Internet Res. (2018) 20(11):e270. 10.2196/jmir.9366 30401664 PMC6246971

[39] Hussain A Tahir A Hussain Z Sheikh Z Gogate M Dashtipour K. Artificial intelligence-enabled analysis of public attitudes on Facebook and twitter toward COVID-19 vaccines in the United Kingdom and the United States: observational study. J Med Internet Res. (2021) 23(4):e26627. 10.2196/26627 33724919 PMC8023383

[40] Kong W Song S Zhao YC Zhu Q Sha L. Tiktok as a health information source: assessment of the quality of information in diabetes-related videos. J Med Internet Res. (2021) 23(9):e30409. 10.2196/30409 34468327 PMC8444042

[41] Sathyanarayana A Joty S Fernandez-Luque L Ofli F Srivastava J Elmagarmid A. Sleep quality prediction from wearable data using deep learning. JMIR Mhealth Uhealth. (2016) 4(4):e125. 10.2196/mhealth.6562 27815231 PMC5116102

[42] Lan K Wang Dt Fong S Liu LS Wong KKL Dey N. A survey of data mining and deep learning in bioinformatics. J Med Syst. (2018) 42:139. 10.1007/s10916-018-1003-9 29956014

[43] Dunn AG Leask J Zhou X Mandl KD Coiera E. Associations between exposure to and expression of negative opinions about human papillomavirus vaccines on social media: an observational study. J Med Internet Res. (2015) 17(6):e144. 10.2196/jmir.4343 26063290 PMC4526932

[44] Hung M Lauren E Hon ES Birmingham WC Xu J Su S. Social network analysis of COVID-19 sentiments: application of artificial intelligence. J Med Internet Res. (2020) 22(8):e22590. 10.2196/22590 32750001 PMC7438102

[45] Corley CD Cook DJ Mikler AR Singh KP. Text and structural data mining of influenza mentions in web and social media. Int J Environ Res Public Health. (2010) 7(2):596–615. 10.3390/ijerph7020596 20616993 PMC2872292

[46] Ranganathan R Mehta V Valkunde T Moustakas E. Topics, trends, and sentiments of tweets about the COVID-19 pandemic: temporal infoveillance study. J Med Internet Res. (2020) 22(10):e22624. 10.2196/22624 33006937 PMC7588259

[47] Griffith J Marani H Monkman H. COVID-19 vaccine hesitancy in Canada: content analysis of tweets using the theoretical domains framework. J Med Internet Res. (2021) 23(4):e26874. 10.2196/26874 33769946 PMC8045776

[48] Li J Xu Q Cuomo R Purushothaman V MacKey T. Data mining and content analysis of the Chinese social media platform Weibo during the early COVID-19 outbreak: retrospective observational infoveillance study. JMIR Public Health Surveill. (2020) 6(2):e18700. 10.2196/18700 32293582 PMC7175787

[49] Ram S Zhang W Williams M Pengetnze Y. Predicting asthma-related emergency department visits using big data. IEEE J Biomed Health Inform. (2015) 19(4):1216–23. 10.1109/JBHI.2015.2404829 25706935

[50] Kuponiyi A Akomolafe OO. Digital transformation in public health surveillance: lessons from emerging economies. Int J Adv Multidiscip Res Stud. (2025) 5:1702–13. 10.62225/2583049X.2025.5.4.4836

